# A systematic review of the impact of stimulus presentation modality on fMRI studies for presurgical mapping

**DOI:** 10.1016/j.ynirp.2026.100412

**Published:** 2026-09-29

**Authors:** Fredrik Brekke, Karsten Specht, Victoria Sherwood, Jakub Wojciechowski

**Affiliations:** a Department of Clinical and Biological Psychology, University of Bergen, Bergen, Norway; b NordicNeuroLab AS, Bergen, Norway; c Mohn Medical and Imaging Visualization Centre, Haukeland University Hospital, Bergen, Norway; d Bioimaging Research Center, Institute of Physiology and Pathology of Hearing, Warsaw, Poland

## Abstract

• FMRI can be valuable for localizing and measuring language lateralization in patients with various pathologies.
• Few studies compare auditory and visual language fMRI paradigms for presurgical mapping, and methods vary considerably.
• Both visual and auditory paradigms show good concordance with other measures and may aid routine preoperative language mapping.

## Graphical abstract

**Figure:**
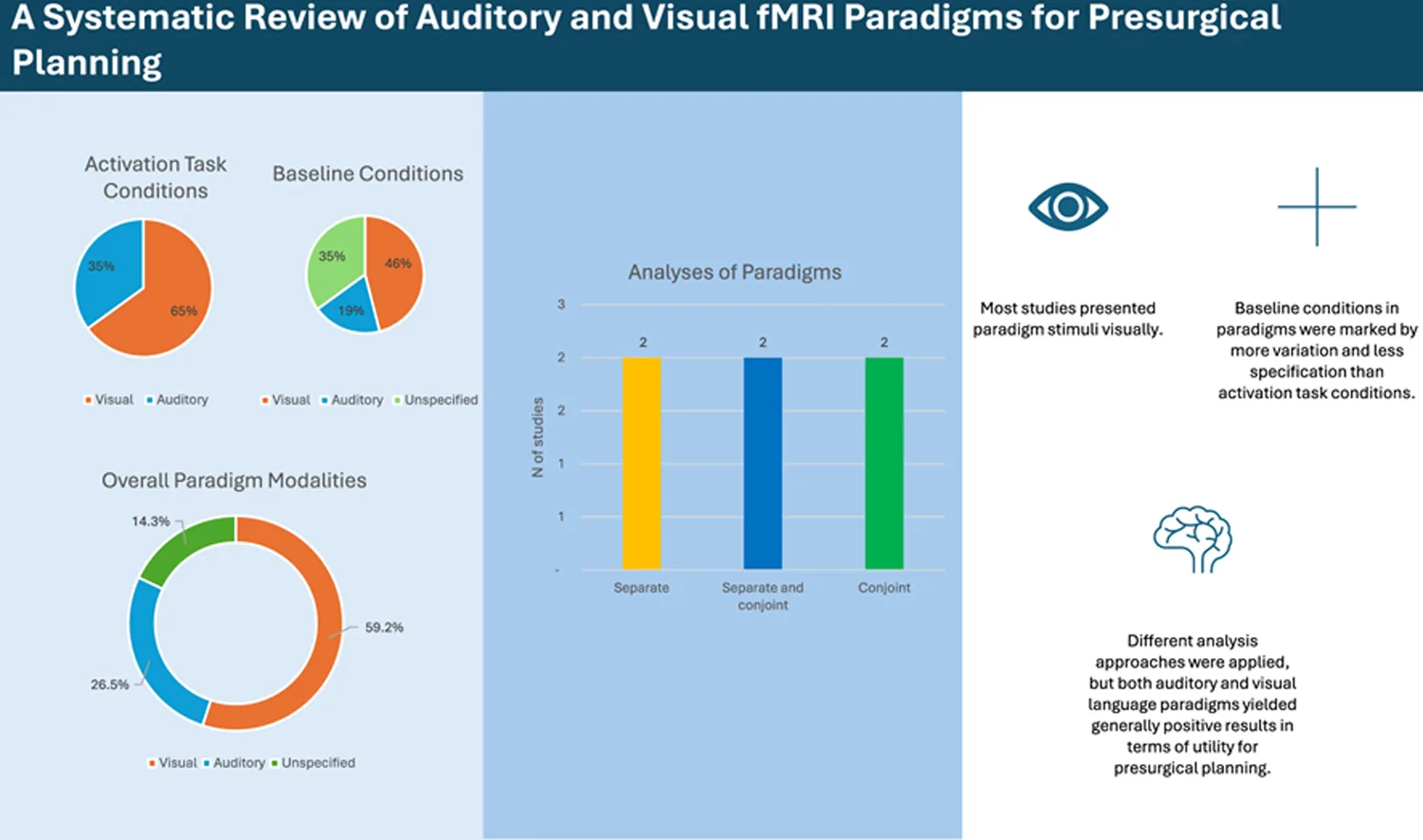

## 1 Introduction

The characteristics of patients considered for brain surgery vary considerably; however, presurgical functional mapping appears to be most commonly applied in patients with neuro-oncological disease (primary or metastatic tumors) and drug-resistant epilepsy (DRE). It has been estimated that about 50 million suffer from epilepsy worldwide, with 20% to 40% being drug-resistant, whereof one third could be surgical candidates (Asadi-Pooya et al., 2022; Samanta et al., 2021; Vaughan et al., 2018). The most common brain tumor types are meningiomas, which are more often benign than malignant, whereas gliomas are the most common malignant brain tumors (glioblastomas are most frequent, followed by low-grade gliomas) (Kim et al., 2024; Lenga et al., 2024; M. Price et al., 2025; Schaff and Mellinghoff, 2023). Malignant brain tumors are typically managed with surgical intervention—either resection or biopsy—depending on tumor location and patient factors, whereas the management of benign tumors is more heterogeneous (Goldbrunner et al., 2021; Weller et al., 2021). Patient selection and surgical planning require careful, individualized risk–benefit assessment, balancing the potential therapeutic gains against the risk of neurological deficits. The aim is to maximize the extent of resection while minimizing the risk of postoperative functional deficits, and ideally improve patient quality of life (Hervey-Jumper and Berger, 2016; Leone et al., 2025). Complete preservation is, however, not always entirely achievable. For example, surgery for temporal lobe epilepsy (TLE) may remove healthy tissue near the epileptogenic zone to improve post-operative seizure control, which, in turn, can cause cognitive deficits (Clusmann, 2008).

Predicting whether neurological function will be preserved or improved requires careful evaluation of preoperative functional status and clinical characteristics. In addition to the prognostic role, presurgical assessment plays a critical role in perioperative risk stratification and in guiding the extent and trajectory of resection (Hervey-Jumper and Berger, 2016; Leone et al., 2025; Massot-Tarrús and Mirsattari, 2022). Presurgical evaluations could include assessments of the lateralization, location and distance of the affected brain tissue to functional areas of the brain (Håberg et al., 2004; Krishnan et al., 2004; Wood et al., 2011), demographic factors and other patient characteristics (Leone et al., 2025; Omisade et al., 2019; Yao et al., 2020), the urgency of brain surgery (e.g., type, grade, and location of pathology, and risk to life and function), the surgical question (e.g., complete/incomplete resection), and the equipment and capacity of the medical center.

There are several techniques that may be utilized to guide, inform, and refine a surgical approach preoperatively and intraoperatively, and to assess the risk of postoperative deficits. Task-based fMRI (tb-fMRI) is currently the most widely used non-invasive technique for presurgical functional mapping and surgical planning in patients with brain tumors and epilepsy, owing to its broad availability, whole-brain coverage, and integration into clinical workflows (Benjamin et al., 2018; Cargnelutti and Tomasino, 2023; Kreidenhuber et al., 2019; Voets et al., 2025). Resting-state fMRI (rs-fMRI) is increasingly being explored as an option to tb-fMRI and integrated into clinical workflows, particularly for patients who are unable to perform tasks, but may be more variable in terms of sensitivity and specificity, and is less standardized than tb-fMRI (see e.g., Abu Mhanna et al., 2025; Boerwinkle et al., 2024; Kumar et al., 2024; Lakhani et al., 2023; Radwan et al., 2024; Specht, 2020 for more details). Since the utility of tb-fMRI can be affected by several factors related to the experimental regime (e.g., stimuli, task, control condition, patient training and instructions, imaging hardware) (e.g., Grill-Spector et al., 2006; Voets et al., 2025), patient (e.g., movement, compliance, medications, engagement, cognitive ability, time of day) (e.g., Specht, 2020; Vaisvilaite et al., 2022), and analysis (e.g., processing steps, analysis decisions, thresholding, choice of software), the repeatability of fMRI has been contested within the surgical community. It seems that the repeatability generally can be trusted (given a consistent approach), however possibly with some variations depending on task type and patient group (e.g., high-grade glioma) (Agarwal et al., 2018; Morrison et al., 2016; Stevens et al., 2016), and it is likely that variations in the fMRI protocol affect localization more than lateralization of a function (Benjamin et al., 2018). Navigated transcranial magnetic stimulation (nTMS) and magnetoencephalography (MEG) are other non-invasive techniques that are being increasingly implemented preoperatively as well (Leone et al., 2025). Moreover, fMRI, nTMS, and MEG may be combined with each other for preoperative planning, taking advantage of the benefits of each (fMRI has superior spatial resolution, nTMS can be used for causal mapping of eloquent cortex, and MEG has superior temporal resolution over fMRI) (Cargnelutti and Tomasino, 2023; Leone et al., 2025; Setlak et al., 2026). Intraoperatively, electrical stimulation mapping (ESM) (also referred to as direct electrical stimulation (DES)) is considered the gold standard for intrahemispheric localization because it allows precise mapping of eloquent cortex, which is possible to do throughout the surgical procedure (Duffau, 2015, 2018). Intracarotid amobarbital (Wada) testing has been a common procedure for functional mapping of epilepsy patients, for which lateralization is more commonly mapped than intrahemispheric distribution of language and memory (Bookheimer, 2007; Stippich et al., 2022) but is currently being replaced by other methods (Massot-Tarrús and Mirsattari, 2022). In general, it is common that both non-invasive and invasive brain mapping techniques are applied for surgical interventions. See, for example, Leone et al. (2025) for more information on non-invasive preoperative techniques and their relation to intraoperative techniques. Discussing other methods than tb-fMRI in more detail goes beyond the scope of the current investigation.

Presurgical fMRI is typically utilized for mapping motor, language, and memory functions (historically more often motor and language functions than memory). Visual and (somato)motor systems can typically be localized consistently by fMRI in expected areas of the brain, with improved sensitivity and specificity for 3T compared to 1.5T (Bookheimer, 2007; García-Eulate et al., 2011; Krasnow et al., 2003; Kuchcinski et al., 2015; Morrison et al., 2016; Silva et al., 2018; Tyndall et al., 2017). Language and memory networks are more widely distributed in the brain and present more complicated cases than motor functions (i.e., more functional neuroanatomical variation). Moreover, there exist various taxonomies of the processing subsystems of language and memory, and their respective expected localization in the brain, however certain regions are more and less expected to be involved in language (see e.g., Benjamin et al., 2017; Hagoort, 2017; Price, 2012; Tremblay and Dick, 2016) and memory (see e.g., Ferbinteanu, 2019; Kim, 2022; Sherman et al., 2024).

A multitude of activation and control tasks have been developed, proposed, and applied over time to deliberately engage essential language and memory systems in the brain while discerning function-specific from function-supporting activity. Despite that, there is no single universally applicable fMRI paradigm that can be used to stimulate and map language or memory, since a single paradigm will not necessarily elicit robust activity in every critical area of the brain in each individual case. Thus, multiple tasks should be applied for presurgical fMRI to avoid misidentification of, and increase confidence in, focal activations associated with language and memory (Benjamin et al., 2018; Black et al., 2017; Buck and Sidhu, 2020; Stippich et al., 2022; Voets et al., 2025). Furthermore, no single set of language paradigms can be applied to every patient either due to variations in clinical aims and/or patients’ abilities to perform the tasks. While recommendations for presurgical language fMRI have emerged, standards for localizing and lateralizing memory are less developed: The clinical applicability of memory fMRI has been reported on, with robust evidence on the utility for mapping verbal memory and predicting outcomes, however, the evidence on non-verbal memory is more limited (i.e., memory mapping follows often a material-specific model focused on verbal and non-verbal systems) (see e.g., Buck and Sidhu, 2020; Doucet et al., 2015; Massot-Tarrús et al., 2019; Robert Powell et al., 2004; Szaflarski et al., 2017; Towgood et al., 2015; Willment and Golby, 2013).

Differences in performance of and brain responses to tasks can occur depending on how they are conveyed (Benjamin et al., 2010; Benjamin et al., 2018; de Guibert et al., 2010). Task stimuli and instructions can, for example, be presented visually via a screen, or audibly via headphones. It has been suggested that it can be valuable to adapt the sensory modality of stimuli for patients with sensory impairments or suit their preferences (Kollndorfer et al., 2013; Voets et al., 2025). Paradigms are, however, more often conveyed visually, which typically leads to higher quality stimuli than audio presentation due to the acoustic noise of the scanner interfering with the presentation of audible content. Furthermore, fewer validated audible paradigms are available for presurgical fMRI protocols compared with visual paradigms, which is problematic when patients have visual impairments or cognitive limitations that reduce the possibility of conveying paradigms visually. Also, the proximity of the primary auditory cortex to Wernicke's area could inherently make auditory language tasks more difficult to interpret than visual (i.e., easier to disentangle visual processing from language processing), which highlights the necessity of appropriate baseline conditions for subtracting basic acoustic processing (Peelle, 2014). Moreover, auditory and visual stimuli are fundamentally different in their characteristics. For example, audible and visible stimuli reflects a qualitatively different relationship to time and space: Unlike visual stimuli, which typically persist and can be re-examined, auditory information is intrinsically sequential and irreversible as information unfolds over time. Consequently, speech perception relies critically on the precise temporal dynamics of the auditory signal, as listeners must discriminate between phonemes that may differ by only tens of milliseconds. Nevertheless, audible paradigms may be conveyed at a different rates to compensate for sensory, attentional, and cognitive capacities (Omisade et al., 2019). Moreover, spatial features in the auditory system is a subordinate dimension compared to the temporal, whereas that is opposite for the visual system (Gori et al., 2026). Furthermore, visual information is remembered better and with more detail than auditory information, if recalled the same day, but auditory memories may be more stable, or just as stable, over time compared to visual memories (Boenniger et al., 2021; Gloede and Gregg, 2019). Thus, it is suggested that visual memory is more susceptible to loss than auditory memory over time, and that the short-term differences are likely more related to the nature of the stimuli, which might demand more attentional resources for auditory than visual stimuli encoding, rather than long-term memory per se (Gloede et al., 2017; Gloede and Gregg, 2019). Nevertheless, it has been indicated that visually presented language paradigms might require an additional attention network, and that visual tasks engage a dorsally-biased attention network, compared to audibly presented language paradigms, which recruits a ventrally-biased network (Kollndorfer et al., 2013; Salmi et al., 2026). This is likely to partially shape the recruitment of language networks during task performance (e.g., Keitel et al., 2020; Kollndorfer et al., 2013), with potential implications for presurgical language mapping in patients with reduced attentional capacity or impairments affecting auditory or visual processing.

Despite modality-specific differences, it is generally acknowledged that there exist modality-invariant language (see e.g., Arana et al., 2020; Drijvers et al., 2025; Fedorenko et al., 2024; Friederici, 2026 for overview and discussions regarding the modality-specific/independent language networks) and memory systems (see e.g., Binder et al., 2009; Park et al., 2025; Patterson et al., 2007; Rugg and Renoult, 2025), which highlights that the same core message can be conveyed regardless of the sensory modality. Although core motor, language, and memory systems are generally seen as supramodal, it remains unclear whether performance on and brain activations to paradigms targeting such functions will be directly affected by the sense that is stimulated, which is particularly valuable to have a clearer understanding of when patients with highly varying characteristics are to be evaluated.

This systematic review aims to investigate the scientific evidence that is available for making decisions about visually and audibly presented paradigms for presurgical fMRI of motor, language, and memory functions. With ongoing advancements in stimulus presentation technologies in the MR environment—particularly for audio delivery—it is important to understand how the choice of sensory modality (or the combination of modalities) may influence the mapping of functional neuroanatomy using tb-fMRI in patients with varying pathologies and lesion locations. Thus, the present systematic review aims to synthesize the existing literature on the impact of sensory modality in presurgical mapping using fMRI. The findings may help clarify the relative effectiveness of auditory and visual paradigms and inform future studies seeking to enhance the applicability of presurgical tb-fMRI across sensory modalities, including both unimodal and multisensory approaches.

## 2 Methods

A systematic review was conducted for records of original studies that have applied comparable auditory and visual fMRI tasks for presurgical mapping of motor, language, or memory functions. The search method used in the present study followed the Preferred Reporting Items for Systematic Reviews and Meta-analyses (PRISMA) (Page et al., 2021).

### 2.1 Search strategy

Articles were identified through a systematic search, which was conducted on April 18, 2025, in Ovid MEDLINE® (All), wherein Embase and APA PsychInfo were included. Specific search terms and MeSH terms for fMRI, auditory, visual, surgery, and motor, memory, and language functions were included in the search, limited to humans, English language (of article), peer-reviewed journals, and non-preprint records (which does not exclude conference papers) (see Supplementary material 1 for full database search).

### 2.2 Selection of records

Records were excluded if fMRI was not used for presurgical mapping, despite applying comparable auditory and visual paradigms on clinical groups or on healthy controls with the aim of instructing clinical fMRI protocols. The rationale for including presurgical fMRI studies only is to ensure that the results indicate how it *is* used and reported for that purpose. Moreover, prioritizing studies that include auditory and visual paradigms in the same study enhances the possibility of directly comparing the impact of sensory modality on the results, compared to studies applying either visual or auditory paradigm versions separately. This decision is related to the high heterogeneity in study (e.g., stimuli, task conditions, population), hardware, and analysis features (e.g., processing steps, quantification approaches) within the field.

Reviews, thesis defense papers, and records without available full text were excluded (see Supplementary material 2 for full description of the protocol). Resting-state fMRI studies were excluded as well. Moreover, the focus of this review is on how different types of sensory information (auditory, visual) can influence neural activation patterns, and the goal is to contribute to the guidance of fMRI paradigm design and task selection for presurgical planning.

Records were assessed for inclusion through blinded co-screening in Rayyan by two independent reviewers (Ouzzani et al., 2016). Reviewer 1 has doctoral-level expertise in neuroscience, with formal training in cognitive science and research experience in cognitive and clinical neuroscience, including applied fMRI for presurgical mapping. Reviewer 2 is a PhD candidate with a master's degree in behavioral neuroscience and training and research experience in cognitive neuroscience. Discrepancies were discussed and resolved in consensus meetings among the four authors. All have relevant academic training and research experience in neuroscience and contributed to reaching final agreement on inclusions and exclusions. Titles and abstracts were assessed in the first stage of the screening process. Articles that met the inclusion criteria were assessed in full text for eligibility. Information from the included records was extracted and synthesized into an Excel spreadsheet. The most relevant study details affecting brain activity in response to tasks are characteristics of participants, task conditions, pre-scan assessments and training, instructions, acquisition, preprocessing, and data analyses. Thus, these were the prominent categories added to the spreadsheet (see Supplementary material 3 for more information on various categories).

## 3 Results

The systematic search identified 509 records initially. Duplicates were removed in Medline (Ovid). 209 of the records were excluded as duplicates. The remaining 300 articles were successfully retrieved. Seven studies met the inclusion criteria (Carpentier et al., 2001; Heilbrun et al., 2002; Metz-Lutz et al., 2000; Ni et al., 2019; Połczyńska et al., 2017; Pouratian et al., 2002; Trimmel et al., 2019). One (Heilbrun et al., 2002) of the seven studies was excluded from the final sample after full-text evaluation due to lacking specification of how fMRI was used for presurgical planning (see Fig. 1 for overview of the review process).

**Fig. 1:**
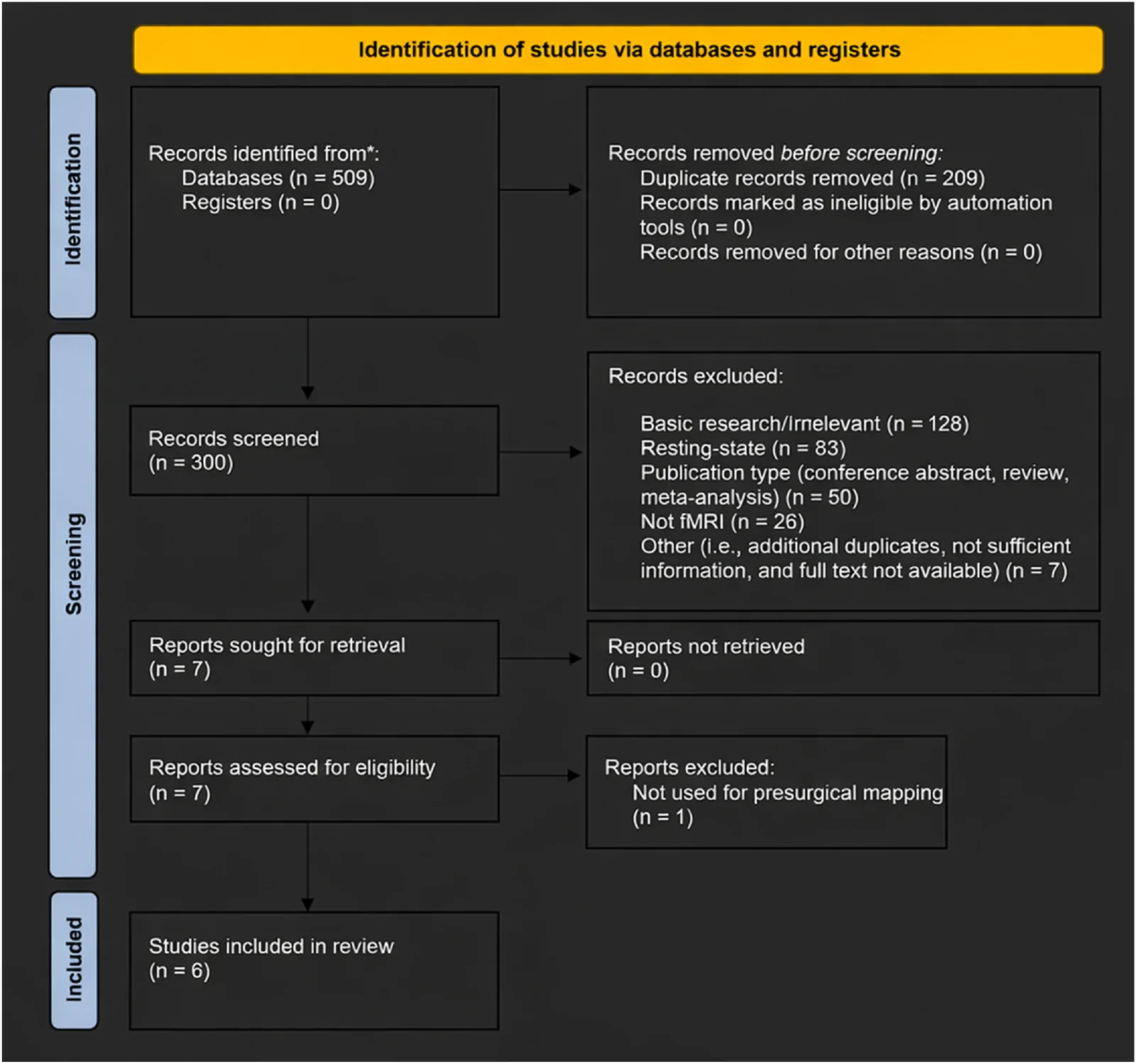
PRISMA flow diagram for the systematic review process illustrating the number of identified, included, and excluded records (Page et al., 2021).

### 3.1 Study characteristics

The age of the participants ranged between 9 and 62 years. Only one study (Ni et al., 2019) reported including patients under 19 years of age. Pouratian et al. (2002) did not report the age of the patients. The number of patients varied from one to 65. Five of the six studies included epilepsy patients (Carpentier et al., 2001; Metz-Lutz et al., 2000; Ni et al., 2019; Połczyńska et al., 2017; Trimmel et al., 2019), three of which included TLE patients exclusively (Metz-Lutz et al., 2000; Połczyńska et al., 2017; Trimmel et al., 2019). One study performed presurgical fMRI in patients with arteriovenous malformations (AVM) and cavernous angiomas (Pouratian et al., 2002). Furthermore, one of the studies was a case-study on a single TLE patient with thalamic infarction, however, the outcomes were compared with two other patients with TLE and hippocampal sclerosis but no thalamic infarction (Metz-Lutz et al., 2000). Epilepsy patients made up the largest group in the Ni et al. (2019) study, but patients with a broad range of pathologies (and cognitive abilities) were included. Additionally to TLE patients, Połczyńska et al. (2017) included sixteen tumor patients with pathology in different locations. The patients in Połczyńska et al. (2017) were characterized by varying levels of education (12-20 years), language deficits, and some had undergone surgery before the study, whereas others had not. Note that only left hemispheric patients were included in the analysis (i.e., right dominant patients, as determined by standard language fMRI tasks (object naming, auditory responsive naming, and visual responsive naming) and/or Wada testing, were excluded (N = 5)). Furthermore, two of the studies compared patients with healthy controls (Carpentier et al., 2001; Trimmel et al., 2019). The groups were relatively matched on age and gender in both studies. The other four studies included surgical candidates only (Metz-Lutz et al., 2000; Ni et al., 2019; Połczyńska et al., 2017; Pouratian et al., 2002) (See Table 1 for an overview).

**Table 1:** Demographic and clinical characteristics.

Study	Number (Sex)	Patient groups	Age	Handedness		Site of pathology	
				Right/Left	Measure	Specific site (pathology)	Right hemisphere/*Left hemisphere*
Połczyńska et al. (2017)	25 (13 F, 12 M)	9 TLE 16 tumors (tumor types = N/A)	Mean = 38.8 (±11.7) years TLE: AO = 24.7 (±12.2) years	20/4 1 Ambidextrous	N/A	E: 9 TL Tumors: 6 TL, 3 FT, 3 FL, 2 TP, 1 PL, 1 FP	E: 1/8 Tumors: TL = 2/4 FT = 2/1 FL = 0/3 TP = 0/2 PL = 1/0 FP = 1/0
Trimmel et al. (2019)	46 P (20 F, 26 M) 19 CTR (9 F, 10 M)	46 TLE	LTLE Mean = 37.5 (±11.2) years AO = 17.8 (±10.7) years D = 13 (29) years RTLE Mean = 38.8 (±11.1) years AO = 20.2 (±14.7) years D = 16 (13.5) years CTR 23-63 years	L TLE: 21/4 R TLE: 19/2 CTR: 17/2	EHI	TLE	21/25
Pouratian et al. (2002)	10 (4 F, 6 M)	7 AVM 3 cavernous angiomas	N/A	All were right-handed	N/A	2 AVM cases with location in the FL and 5 TP cases Size range = 4 mm – 3 cm	N/A
Ni et al. (2019)	40 (16 F, 24 M)	Largest group: 16 E patients with different epileptogenic foci. Other pathology types: encephalomalacia (4), tumor (1), artery occlusion (2), atrophy (7), schizencephaly (1), ventriculomegaly (2), focal cortical dysplacia (5), No visible lesion (1), space occupying LaV (1)	Mean = 25.05 (±9.05) years D = 13.2 (±7.47) years	36/4	N/A	5 L T 5 L FTP 4 L F 1 R F 2 L LaV 2 L TO 6 LHemi (1 with EF in LPO and 1 with EF in LTP) 1 Cerebellar 1 L C 1 L OL 1 L PL 1 L FT 1 L FTPO 1 L FP 1 With no lesion 7 additional L but with inappropriate use of abbreviations (i.e., letter abbreviations were not clearly defined).	2/38
Carpentier et al. (2001)	10 P (5F, 5M) 10 CTR (4F, 6M)	10 E	P Mean = 37.9 (24-51) years D = 18.9 (1-41) years CTR Mean = 32.3 (19-62) years	P 7/2 1 Ambidextrous CTR 8/2	N/A	2 Left PL (cortical migration disorder, porencephaly) 1 Left FL (dysplasia) 2 Left hippocampal (1 with Sylvian as well. Ischemia, atrophy) 2 Left ITL (lesion, DNET) 1 Left fusiform (cavernoma) 1 Left TP (Sturge-Weber syndrome) 1 Left posttraumatic focus (normal MRI)	LH
Metz-Lutz et al. (2000)	1 (F)	1 TLE	49 years	1/0	N/A	1 L TLE and Left thalamic infarction post-surgery	LH

*AO = Age of onset. AVM = Arteriovenous malformation*. *C = Cerebellar CTR* = *Healthy control*. *D = Duration. E = Epilepsy*. *EHI = Edinburgh Handedness Inventory*. *ESM = Electrocortical stimulation mapping. F = Female. FL = Frontal lobe. FP = Frontoparietal. FT = Frontotemporal. ITL = Inferior temporal lobe. LaV = Lateral ventricle. L = Left. LH = Left hemisphere. M* = *Male*. *N/A = Not available*. *OL = Occipital lobe. PL = Parietal lobe. R = Right*. *TL = Temporal lobe. TLE* = *Temporal lobe epilepsy*. *TP = Temporoparietal*.

### 3.2 Visual and auditory paradigms

For the activation task conditions, 15 of 23 (65%) were visual, whereas eight of 23 (35%) were auditory. Naming paradigms were most frequently applied (10/23; 44%) of the activation task conditions. These were presented through pictures in four and text in three of the visual task versions (7/10), and via audio in three of the acoustic versions (3/10).

Baseline conditions were characterized with more variation and were reported with less details than activation conditions. The most frequent baseline conditions of those reported were (unspecified) rest (9/26; 35%) and rest with crosshair fixation (8/26; 31%). Lastly, Metz-Lutz et al. (2000) reported the only study to present the majority of conditions audibly (4/6). Note that fewer task conditions than baseline conditions were applied (23 compared to 26), however, it was only Trimmel et al. (2019) that applied multiple baseline conditions in their paradigms. Moreover, the sensory modality was not reported for 9 of 26 (35%) baseline conditions.

The included studies applied multiple paradigms (2-6). Considering all of the task conditions (activation and baseline), 29 of 49 of the stimuli were reported to be visual (59.2%) and 13 of 49 were auditory (26.5%) (see Table 2 for overview of the specific paradigms and sensory modalities).

**Table 2:** Studies with auditory and visual paradigms included in presurgical fMRI.

Study	Activation tasks	Modality	Baseline conditions	Modality
Połczyńska et al. (2017)	Object naming		Rest	N/A
	Visual responsive naming		Rest	N/A
	Auditory responsive naming		Rest	N/A
	CYCLE-N		Rest	N/A
Trimmel et al. (2019)	Overt auditory naming		Reversed speech AND Crosshair fixation	AND
	Overt picture naming		Scrambled pictures, Blurred cartoon faces, AND Crosshair fixation	
	Verbal fluency		Crosshair fixation	
Pouratian et al. (2002)	Visual object naming		Rest	N/A
	Word generation		Rest	N/A
	Auditory responsive naming		Rest	N/A
	Visual responsive naming		Rest	N/A
	Sentence comprehension		Rest	N/A
Ni et al. (2019)	Text reading		Crosshair fixation	
	Word comprehension		Crosshair fixation (Green cross)	
	Verb generation		Crosshair fixation	
	Visual object naming		Crosshair fixation	
	Sentence reading		Crosshair fixation	
	Auditory comprehension		Scanner noise	
Carpentier et al. (2001)	Auditory language (decision) task		Tone-decision	
	Visual language (decision) task		Cross-match line decision	
Metz-Lutz et al. (2000)	Verb generation		Mental word repetition	
	Auditory word listening		Reversed auditory word listening	
	Visual lexical decision		Unreadable strings of characters	

*= Visual stimuli*.  = *Audible stimuli*. *N/A* = *Not available*.

### 3.3 Functional domain

The six studies assessed language functions, each with individual aims: Połczyńska et al. (2017) assessed whether adding grammatical demands to a language task could improve language mapping, compared to a standard clinical fMRI protocol (i.e., three different naming tasks). Trimmel et al. (2019) assessed how well language fMRI paradigms can be used to predict postsurgical naming decline in TLE patients. This was the only study that demanded overt responses on some of the tasks. Pouratian et al. (2002) evaluated the concordance between fMRI and ESM for mapping language in patients with cerebrovascular malformations (i.e., AVM and cavernous angioma). Ni et al. (2019) assessed the validity and utility of a preoperative fMRI procedure with six language tasks, which were adapted to individual abilities and progressively applied. FMRI activations and laterality indices (LI) were compared with ESM. Carpentier et al. (2001) evaluated the utility of fMRI for mapping language in epilepsy surgery candidates by comparing the fMRI maps with electrophysiology measures and the Wada test. Finally, Metz-Lutz et al. (2000) reported on the role of the thalamus for language processing and performance on verbal tasks through pre-and post-surgical fMRI and cognitive tests. They found that changes in fMRI patterns were concomitant with changes in performance on the cognitive test batteries.

### 3.4 Analytical approaches

All studies performed some quantitative analysis of BOLD activation areas, either for direct comparison of specific areas between individuals or groups, or for calculation of laterality indices. The approaches and decisions for calculating activation maps varied between the studies. Five of six studies used a whole-brain approach, where mean or peak activation values (t-value or z-value) across the brain were used as metrics for comparison (Carpentier et al., 2001; Metz-Lutz et al., 2000; Ni et al., 2019; Pouratian et al., 2002; Trimmel et al., 2019). Carpentier et al. (2001) used both a whole-brain and a region-of-interest approach to compare laterality scores between a control group and a patient group. Only Połczyńska et al. (2017) used a ROI approach alone. Note that the two ROI approaches differed in size; Połczyńska et al. (2017) applied fixed 5 mm radii for the spheres, whereas Carpentier et al. (2001) had varying surface sizes ranging from 1.8 cm^2^ to 7.8 cm^2^, depending on the region. Furthermore, task-responses were quantified using different BOLD signal metrics (mean or peak), however, the method of calculation was not reported in three studies (Metz-Lutz et al., 2000; Ni et al., 2019; Pouratian et al., 2002). Most studies performed both individual and group-level quantifications, whereas Carpentier et al. (2001) performed analysis on group-level only and Metz-Lutz et al. (2000) on individual-level only. Most of the studies included some sort of preparation and assessment prior to scanning (5/6). There were differences in whether the studies performed conjoint (Połczyńska et al., 2017; Pouratian et al., 2002), separate (Metz-Lutz et al., 2000; Trimmel et al., 2019), or both separate and conjoint analyses (Carpentier et al., 2001; Ni et al., 2019) for task activations, whether laterality indices (LIs) were measured (50% calculated LIs), and how significance was determined (i.e., thresholds and corrections). Lastly, some form for participant training and assessment ahead of scanning was performed in all but one study (see Table 3 for full overview).

**Table 3:** Functional domain, training and assessment, and analysis parameters.

Study	Functional domain	Preparation	Acquisition	Data analysis					
			Basic sequence parameters	Approach	BOLD-Signal	Level	Task activations	LIs	Threshold (Criteria)
Połczyńska et al. (2017)	Language	Y	Siemens Allegra head-only 3 T. TR = 2500ms, TE = 35ms, Res = 3.1 mm^3^.	ROI (N = 18, 9 in each hemisphere; 5 mm radii. Four anterior (BA 44, BA 45, BA 47 and the anterior STG) and five posterior (the posterior MTG, posterior STG, anterior SMG. Posterior SMG, and AG). Taken from a Brodmann's area atlas and from FSL's Harvard-Oxford Cortical Atlas.	Mean	Individual and Group	Conjoint	N	Fixed (Cluster; Z > 2.3, p = 0.05 correction)
Trimmel et al. (2019)	Language	Y	3 T N/A	Whole-brain	Peak	Individual and Group	Separate	Y	Fixed (p < 0.05, FWE-corrected)
Pouratian et al. (2002)	Language	N/A	GE 3T. TR = 2500ms, TE = 45ms, Res = 4 mm^3^	Whole-brain	N/A	Individual, Subgroup, and Group	Conjoint	N	Fixed (N/A)
Ni et al. (2019)	Language	Y	Siemens Prisma 3T. TR = 2000ms, TE = 30 ms	Whole-brain	N/A	Individual and Group	Separate and Conjoint	Y	Individualized
Carpentier et al. (2001)	Language	Y	GE Signa 1.5T. TR = 1500ms for the auditory task and TR = 1848ms for the visual task, TE = 50ms	Whole-brain and ROI (N = 5; 1.8 cm^2^ - 7.8 cm^2^: manually defined; Right/Left BA 22, Left BA 44 and 45, and Right Broca). Defined manually on axial slices from volumes aligned to the Montreal Brain Atlas (−15° from horizontal in Talairach space, parallel to BA 22).	Mean	Group	Separate and Conjoint	Y	Fixed (t > 1.5 on both tasks)
Metz-Lutz et al. (2000)	Language	Y	Bruker system 2T. TR = 5000ms, TR = 43ms, Res = 4 mm^3^	Whole-brain	N/A (Voxelwise)	Individual	Separate	N	Fixed (Cluster correlation >0.5 and > 0.3)

*AG = Angular gyrus. BA = Brodmann area. FWE = Family-wise error. LI* = *Laterality index*. *N* = *No*. *N/A* = *Not available*. *MTG = Middle temporal gyrus. Res = Resolution. ROI* = *Region of interest. SMG = Supramarginal gyrus. STG = Superior temporal gyrus. T = Tesla. TE = Echo time. TR = Repetition time. Y* = *Yes*.

### 3.5 Main findings on sensory modality impacts

Generally, the results from the studies indicated that fMRI can be useful for localizing and lateralizing language in patients with various pathologies. Further, both auditory and visual language paradigms can be useful to do so, however, visual stimuli may lead to more robust results (Carpentier et al., 2001; Trimmel et al., 2019). Moreover, auditory and visual language fMRI activations showed good concordance with ESM (Carpentier et al., 2001; Ni et al., 2019; Pouratian et al., 2002) and the Wada test (Carpentier et al., 2001). Two of the studies also found that task sets with visual and auditory paradigms can be valuable for assessing and predicting postoperative outcomes (Metz-Lutz et al., 2000; Ni et al., 2019; Trimmel et al., 2019).

Regarding auditory versus visual task activations specifically, Trimmel et al. (2019) reported whole-brain group-level maps for each task, which were of combined maps of left TLE (LTLE), right TLE (RTLE) patients and healthy controls (CTR). The predictive value of LIs from each task for naming decline was reported as well. A receiver operating characteristics (ROC) analysis on LTLE patients and prediction of significant naming decline yielded an area under the curve (AUC) of 0.82 and positive predictive value (PPV) of 58% with 100% sensitivity and 58.3% specificity for the auditory naming task. The picture naming task showed an AUC of 0.94 and with a PPV of 91.6% with 100% sensitivity and 92% specificity. Furthermore, they found that LIs of auditory and visual naming task activations were better predictors of naming decline than other parameters (e.g., hippocampal volume, surgery type, preoperative naming scores, age, etc.).

Ni et al. (2019) reported patient-by-patient p-value adjustments and average LIs for each task, and examples of task activations to each task in-text. They found overlap in LIs across tasks in the majority of patients (97.5%; 72.5% with both Broca's and Wernicke's). Carpentier et al. (2001) performed both separate and conjoint analyses of activation areas. This indicated modality-specific and modality-independent activations to auditory and visual language comprehension involving processing of semantic and syntactic information. Their study found that the visual language task was more lateralizing in the control group than in the patient group. In the patient group, the most lateralizing results were found in the combined activation maps of the visual and auditory language task. For the Wada-fMRI comparison, even where the visual task activations corresponded better with the Wada and neuropsychological assessments than the auditory task, the highest agreement was found when analyzing the conjunction of auditory and visual task results. Lastly, Metz-Lutz et al. (2000) reported task-dependent activations and task-related changes after a thalamic infarction, compared with two matching patients without the thalamic infarction. The results suggested that auditory and visual lexical processing may be differentially impacted by such damage. The rest of the studies did not assess task- and modality-dependent/independent activations (see, however, Table 4 for a more detailed overview of the results from each study, which places the results on the sensory modalities in the context of the primary aims and main findings in the papers).

**Table 4:** Primary aims and main findings from each study.

Study	Primary aims	Main findings	
		Task activations	Laterality
Połczyńska et al. (2017)	Assess whether a complex sentence-level task with increased demands of grammar comprehension and production (CYCLE-N) adds to a standard lexico-semantic clinical fMRI protocol among LH patients.	Group results - LH: Greater mean percent signal on all CYCLE-N tasks in posterior regions compared with all Standard tasks. Greater activity in left AG with CYCLE-N (negative for standard). Greater activity in BA 47 on standard tests. Group results - RH: Greater activity in anterior STG, posterior STG, and AG on CYCLE-N. Individual results - LH: Greater activations in 10 patients on CYCLE-N. Greater activations in 3 patients on standard tasks. Individual results- RH: Greater activations in 12 patients on CYCLE-N. Greater activations in 2 patients on standard tasks.	Increased bilateral hemispheric ROI activity during CYCLE-N compared with the standard tests.
Trimmel et al. (2019)	Develop and test language fMRI approaches that may be used for predicting postsurgical naming decline in TLE patients.	Task-activations across groups Overt AN = left posterior ITG, left posterior MTG, left FG, left IFG, left SFG, and SMA Overt PN = left FG, left SMA, left MOG, bilateral cuneus, right IOG, and right cerebellum. Covert VF = left IFG, left precentral gyrus, left SMA, right lingual gyrus, left IPL, left IOG, bilateral MOG, and bilateral cerebellum. Naming decline AN = In LTLE patients, stronger activations in left posterior ITG and left IFG were related to greater decline. PN = In LTLE patients, stronger activations in left FG, left MOG, and right cerebellum were related to greater decline. VF = No related activations. In LTLE patients, ROC revealed a PPV of 58.3% with 100% sensitivity and 58% specificity for AN, and a PPV of 91.6% with 100% sensitivity and 92% specificity for PN.	Naming decline AN = LH lateralization was correlated with greater naming decline in LTLE, and left LIs were the best predictor for naming decline. PN = Greater naming decline for both LH lateralization in LTLE patients and RH lateralization in RTLE patients. LIs were the best predictor for decline, but smaller contralateral preoperative hippocampal volume predicted decline significantly in LTLE as well. VF = The only significant relation with naming decline was for left-sided lateralization.
Pouratian et al. (2002)	Assess the concordance between fMRI and ESM on AVM and cavernous angioma patients in order to instruct utility of fMRI for presurgical mapping.	fMRI activations corresponded consistently with ESM across the individual patients, yielding fMRI as a good predictive tool for intraoperative mapping, particularly with 5 mm radius and a strict overlap criterion. Activations in close proximity to pathology sites overlapped consistently between fMRI and ESM as well. Expected activations were found in left IFG and left posterior STS. Production Overall, across different radii and matching criteria, the strongest association between fMRI and ESM was near the IFG. True-positive rates for fMRI was 87.5%. In patients with AVMs, sensitivity and specificity values demonstrate that the usefulness of fMRI is primarily limited to the frontal lobe, despite significant associations with ESM in both frontal and temporoparietal areas. In patients with cavernous angiomas, the associations were significant in frontal lobes, however, restricted to narrow radii. Comprehension Overall, across different radii and matching criteria, the strongest association between fMRI and ESM was near temporal and parietal lobes. True-positive rates for fMRI was 65.8%. In patients with AVMs, sensitivity and specificity values are comparable to the general patient population in this study. In patients with cavernous angiomas, the association was consistent in both frontal and temporoparietal lobes.	LH language localization.
Ni et al. (2019)	Assess the validity and utility of a preoperative fMRI procedure containing six language tasks. Task activations and LIs were compared with ESM.	fMRI - ESM concordance Mappings yielded a high concordance in 80% of the patients, particularly with left-lateralized fMRI activations. RH was not assessed with ESM. Concordance between fMRI and ESM was moderate in 15% of the patients, despite being deemed right-lateralized by fMRI since positive areas were identified by both ESM and fMRI in the LH. There was low concordance between ESM and fMRI in one patient (5%), wherein fMRI showed no LH activations, but positive LH sites were identified by ESM in the left temporal lobe (again, RH was not assessed by ESM). Overlap in fMRI activations across tasks 39 of 40 (97.5%) showed overlapping activations across tasks: 29 (72.5%) in both Broca's and Wernicke's area, and 10 (25%) in Broca's only. The LIs in 37 of the 39 patients (94.9%) with overlapping activations in Broca's and Wernicke's were consistent with their determined lateralization.	LIs of Broca's area (BA 44, 45, 9, and 46) and Wernicke's area (BA 21, 22, 39, and 40). 17 patients showed LH language lateralization. 18 patients showed RH language lateralization. 5 were bilateral. Lateralization was not a preoperative step for selecting patients to surgery.
Carpentier et al. (2001)	Assess the concordance between fMRI, electrophysiology measurement, and the Wada test for mapping language functions in epilepsy surgery candidates.	fMRI - CTR The VL Task and AL Task both activated left IFG: BA 44 were more frequently and strongly activated in the LH during the VL Task (9/10) compared to AL Task (2/10). Both activated BA45 (10/10). SMA was activated more frequently in VL Task than AL Task (8/10 compared to 3/10). Both task versions activated BA 22 robustly (10/10). Bilateral activations of nearby sites (BA 41, 42, and posterior BA 22) were seen more frequently for the AL Task. Bilateral MTG (BA 21) were found in all patients for the AL Task but only left-sided for the VL Task. FuG was active in 7/10 during the VL Task. Left SMG (BA 40) was active in the VL Task only. fMRI - P The strength of activations were lower than in the CTR group. Activations for both tasks (and conjunction) were found in the IFG (BA 44 and 45) and bilaterally in STG (BA 22) (right only for left-handed patients), MTG (BA 21), and ITG (BA 20). IFG activations were more bilateral than in the CTR group as well, and more frequently right-sided. Left FG activated in 60%, mostly by the VL Task. Right SMG and left SMG were activated in 4/10 during the VL Task. Cortical stimulation vs. fMRI Activations were fully concordant (15/15 regions). All of the regions identified by fMRI (71) could not be assessed by stimulation due to limited brain coverage (15).	fMRI – CTR VL Task was more lateralizing than AL Task for both a whole-brain and a ROI approach. Activity intersections between the tasks were most lateralizing. fMRI – P VL Task was not significantly more lateralizing than AL task in the patient group. Activity intersections between the tasks were most lateralizing. Significant difference between right-handed CTR and right-handed P for both tasks individually and the conjunction of them (whole-brain and ROI). Wada vs. fMRI Consistent in 8/10 patients for whole-brain, and 9/10 when BA41/42 was excluded. Generally, comparable for all but two patients.
Metz-Lutz et al. (2000)	Assess the role of thalamus for language processing and performance on verbal tasks by pre-and postsurgical tests.	Silent verb generation Pre-thalamic infarction: Left DLPFC (BA 46-47) and visual cortex (BA 17-18) Post-thalamic infarction: Increased activity in frontal regions (BA 45,46, and 47), and activations in right frontal regions and left MTG (BA 21). Real auditory word listening Pre-thalamic infarction: Left MTG and IFG. Post-thalamic infarction: No more activity was found. Visual lexical decision Pre-thalamic infarction: Bilateral (predominately left) DLPFC (BA 45-46), left SOG and ACC (BA 31). Post-thalamic infarction: Reduced overall activity, except for in left CG (BA 31). Changes in activations from pre- and post-thalamic infarction was concomitant with impaired behavioral performance on the three verbal tasks (i.e., increased false recognitions of words, difficulties finding words, and increased response times and errors).	N/A

ACC = Anterior cingulate cortex. AG = Angular gyrus. AL = Auditory language. AN = Auditory naming. AVM = Arteriovenous malformation. BA = Brodmann area. CG = Cingulate gyrus. CTR = Healthy control. DLPFC = Dorsolateral prefrontal cortex. ESM = Electrical stimulation mapping. FG = Fusiform gyrus. fMRI = Functional magnetic resonance imaging. IFG = Inferior frontal gyrus. IOG = Inferior occipital gyrus. ITG = Inferior temporal gyrus. LH = *Left hemisphere*. LI = Laterality index. LTLE = Left temporal lobe epilepsy. MOG = Middle occipital gyrus. MTG = Middle temporal gyrus. N/A = Not available. P = Patient. PN = Picture naming. PPV = Positive predictive value. ROC = Receiver operating characteristics. ROI = Region of interest. RTLE = Right temporal lobe epilepsy. SFG = Superior frontal gyrus. SMA = Supplementary motor area. SMG = Supramarginal gyrus. SOG = Superior occipital gyrus. STG = Superior temporal gyrus. STS = Superior temporal sulcus. TLE = Temporal lobe epilepsy. VF = Verbal fluency. VL = Visual language.

## 4 Discussion

The primary purpose of this systematic review was to investigate the existing scientific evidence on auditory and visual alternatives of fMRI tasks that are applied for presurgical brain mapping and planning. A total of 300 papers were retrieved from the systematic search, and six of the identified papers were included. In these, task conditions were presented visually 59.2% of the time and audibly 26.5% of the time. For the remaining 14.3% of the task conditions, which were baseline conditions, the sensory stimulation modality was not specified. The six studies used fMRI for assessing language functions preoperatively, mostly with comparison to some other measurement (i.e., ESM, Wada, and/or neuropsychological assessments).

Despite the fact that few studies met the inclusion criteria, the included studies report similar findings to those commonly found throughout the fMRI literature: For example, reports from epilepsy centers and surgical programs share similarities with the findings in this investigation – namely that two or more paradigms are primarily used; resting conditions are the most frequent baseline conditions (but with variability between tasks and centers, and occasional lack of specific information); stimuli are most often visual; patients typically receive the chance to practice tasks and are assessed prior to scanning; and there are variations to whether thresholds are uncorrected or corrected (Bargalló et al., 2020; Benjamin et al., 2018).

### 4.1 Functional domain

That the identified studies meeting the inclusion criteria were all language fMRI studies, and not motor or memory fMRI, was not surprising. Indeed, fMRI is currently most commonly applied for language mapping pre-surgery. With regards to presurgical motor cortex localization, regions can reliably be identified with fMRI, but nTMS has the highest accuracy for motor mapping (Leone et al., 2025). Thus, it is normally not necessary to include both auditory and visual task versions in the same protocol to improve motor mapping, unless visual impairments affects that decision. Moreover, even if motor tasks are typically conveyed visually, adapting motor tasks to be audibly presented would often not require much adaptation (e.g., from “Start” and “Stop” instructions via text to audio). Despite that, audibly cued motor tasks may provide some meaningful differences to visually cued motor tasks (e.g., Jäncke et al., 2000; Witt et al., 2008), which could be relevant to consider, depending on the patient's case. Nevertheless, motor processing is less involved with auditory and visual networks than language (if excluding speech). Language is more inherently linked with the modality, in terms of seeing (reading or looking at objects) and listening being the main ways in which language is conveyed and produced. Thus, there is another level of complexity related to the decision of sensory stimulation modality for language compared to motor mapping. When it comes to presurgical memory fMRI, an optimal protocol has not yet been established in the field, and the number of studies published on auditory and visual memory mapping in this context might be affected by that. Moreover, the field appears to have been more focused on investigating and establishing a material-specific model (i.e., verbal and visuospatial memory in contralateral hemispheres) than on impacts of modality-specific/independent processes.

Even if memory or motor functions were not of primary interest in the reviewed studies, memory and motor processes are often involved in certain aspects of language. Indeed, verbal memory is an important feature for performance on several of the language tasks (e.g., lexical and semantic retrieval on naming tasks). Moreover, language-motor performance can be involved during tasks as well. For example, overt responses to task-stimuli were included in the Trimmel et al. (2019) study for the auditory and visual naming tasks. Overt language responses require motor execution and control, which may be bilaterally activated (Berro et al., 2021), whereas covert language responses require motor planning and simulation of speech, which produces comparable activations to overt task versions, and can improve lateralization (Partovi et al., 2012).

### 4.2 Paradigms

All of the paradigms were language paradigms, however, with varying focus and demands on linguistic processing. Most tasks could be categorized as language generation (word generation, naming tasks), comprehension (sentence/word, reading/listening), or decision (lexical, semantic, syntactic) tasks. In short, generation tasks put emphasis on language output and retrieval (production); comprehension tasks put emphasis on the encoding and decoding of input and integration of linguistic information (understanding); and decision tasks put emphasis on evaluation of language content (metalinguistic awareness, linguistic integration). Word-level tasks are often strongly lateralizing and more confined to the frontal lobes than the temporal lobes, whereas sentence-level tasks normally activate a broader language network in both the frontal and temporal lobes. Simple sentence-level tasks can be less lateralizing than word-level tasks (e.g., reading or passive listening), but sentence comprehension tasks that are more semantically and/or syntactically demanding and actively engaging (e.g., sentence-completion) are often strongly lateralizing as well (Bradshaw et al., 2017; Voets et al., 2025).

Naming tasks were the most applied language tasks among the reviewed studies. Both auditory and visual (picture) naming versions have been reported elsewhere to robustly activate temporal lobe regions of interest in LTLE and RTLE patients (Gonzálvez et al., 2016), however, picture naming might be less lateralizing and less sensitive to regions typically associated with the distributed frontotemporoparietal language network (i.e., more sensitive to a fronto-occipital network) compared to the audible naming version (Gonzálvez et al., 2016; Trimmel et al., 2019; Wilson et al., 2017). Most of the samples in this review were dominated by epilepsy patients. TLE patients are often marked with dysnomia (i.e., naming difficulties) (Gonzálvez et al., 2016; Hamberger, 2015; Swanson et al., 2007). Thus, applying naming tasks in TLE patients is somewhat paradoxical. An important reason for applying these tasks is that they can help indicate whether deficits are likely due to the hemispheric language dominance or not (Thomas et al., 2023), which has been shown valuable for predicting postoperative outcomes (Bonelli et al., 2012). Furthermore, Trimmel et al. (2019) indicated that activations derived from both an auditory and a visual (picture) naming tasks can be valuable for predicting naming decline in LTLE patients. This was not found to be the case in patients with RTLE due to low occurrence of naming decline in that group. Indeed, naming decline is less prevalent in RTLE patients than LTLE, particularly when the dominant hemisphere for language is the left hemisphere (Bonelli et al., 2012). Studies reported here have indicated that both auditory and visual paradigm versions can be included in task sets to map language functions, however, the number of and the reporting in the studies makes it generally difficult to make clear conclusions about or suggestions to their applicability.

#### 4.2.1 Sensory modality

It has been indicated from ESM studies that auditory responsive naming activates language areas located anterior to visual responsive naming tasks (Hamberger et al., 2001, 2007). Furthermore, sparing of regions associated with auditory naming was found to be related to post-operative naming outcomes, whereas sparing the visual naming areas did not prevent post-operative naming decline (Hamberger et al., 2005; Swanson et al., 2007). This points to the usefulness of including an audible language paradigm for language mapping in TLE patients, and highlights modality-dependent effects for naming (Ekert et al., 2021; Hamberger et al., 2001, 2005). Detection of regions involved in auditory language processes with fMRI is, however, strongly influenced by the baseline task that is applied. Using resting conditions to auditory tasks typically leads to bilateral activations in the temporal lobe (i.e., near the Heschl's gyrus, and might expand along the perisylvian sulcus), whereas applying an auditory baseline task designed to subtract low-level auditory processing (e.g., tone decision, backwards speech) may produce a more interpretable map in terms of language processes (Niskanen et al., 2012; Voets et al., 2025). Having an appropriate auditory baseline condition for the activation task is also not necessarily enough to avoid bilateral activity patterns in the contrasted and thresholded maps; the acoustic noise of the MR scanner can potentially lead to activation of supporting brain mechanisms during task performance to compensate for the degradation of auditory stimuli and increased requirement of attention due to the background noise (Peelle, 2014).

Carpentier et al. (2001) indicated more bilateral activations of the audible task compared to the visual task, but this difference was primarily evident for healthy controls and not the patients, which showed increased bilaterality on both task versions. Further, Carpentier et al. (2001) reported both modality-specific and modality-independent activations, and that a combination of audible and visual paradigms provided the highest laterality scores when input-independent areas were highlighted, and greater agreement with the Wada and neuropsychological findings when combined visual and auditory results were applied than with the modalities alone. Indeed, other reports suggest that using both audible and visual language paradigms can decrease LI variability and improve detection of frontal and temporal language regions; there is more variability of language activations in the temporal lobe for visual compared to audible presentation, whereas the opposite tendency is found for the inferior frontal gyrus (Ekert et al., 2021; Niskanen et al., 2012; Suarez et al., 2008). Also, Omisade et al. (2019) reported promising utility of a composite auditory paradigm, designed for patients with visual impairments and cognitive limitations (however, only tested on healthy controls), as brain activations to the paradigm showed near-perfect agreement with a “standard” visual paradigm for LIs and hemispheric dominance determination. Ni et al. (2019) reported great overlap in Wernicke's and Broca's area across tasks (5 visual and 1 auditory), which at least suggests that overlapping results in core language regions can likely be found between multiple tasks and with both sensory modalities. Moreover, Trimmel et al. (2019) reported a more left-lateralized fronto-temporal language network for the auditory task, whereas the picture naming (visual) task activated a more bilateral fronto-occipital network, suggesting modality-specific impacts on engagement of linguistic operations. Note that the results in Trimmel et al. (2019) were derived from group-based analyses where data from both healthy controls and patients were combined.

The limited basic and clinical research literature suggests that a combination of audible and visual language paradigms might reduce variability in the detection of core language regions, improve lateralization, and highlight modality-dependent differences that can be clinically relevant (e.g., in TLE patients). That being said, based on the currently available literature, it is difficult to provide a clear conclusion on the impact that the sensory modality has on preoperative localization and lateralization of language using fMRI. Nevertheless, because language deficits may occur either within a specific sensory modality or across multiple modalities, it is likely that language paradigms presented through different stimulation modes engage both modality-dependent and modality-independent neural processes, which may be of clinical relevance. For example, patients with receptive aphasia may exhibit severely impaired auditory comprehension despite relatively preserved reading abilities. Similarly, some patients may retain intact hearing and speech production while being unable to recognize the meaning of spoken words (auditory verbal agnosia), whereas others may demonstrate multimodal semantic impairments affecting comprehension of speech, non-verbal sounds, pictures, and written text alike. Observations such as these suggest that language-network recruitment may vary as a function of both sensory modality and the underlying nature of the linguistic deficit. Indeed, tailoring task modality to the patient's neuropsychological profile may improve the likelihood of capturing preserved language function, and discrepancies between modalities may themselves be informative about language organization or impairment. Moreover, the selection of language paradigms in presurgical fMRI should account for the surgical objective as well as the patient's sensory, attentional, and cognitive abilities to adequately perform a particular task. Consequently, the availability of validated preoperative language fMRI paradigms that can be administered in both audible and visual formats may facilitate more individualized and clinically sensitive approaches to presurgical language mapping, but this remains a tentative stance.

### 4.3 Quantification

Two surveys of epilepsy centers not included in this review reported on thresholding; more than half (79%) of the epilepsy centers reported used individualized thresholding (i.e., “dynamic” thresholding) in one report (Benjamin et al., 2018), whereas the other indicated that fixed thresholds were applied in more than half (57%) of the centers (Bargalló et al., 2020). Note that dynamic thresholding involves exploring the impact that various threshold values have on activation maps (typically from low to high), which is an approach to optimize detection of task- and subject-specific activations. Of the reviewed studies, only one study (Ni et al., 2019) applied individualized thresholding. Dynamic thresholding is a recommended approach for mapping of individuals since activation magnitude and signal-to-noise levels can vary between individuals and specific tasks (Morrison et al., 2016; Nadkarni et al., 2015; Pillai and Zaca, 2011; Rutten et al., 2002; Stevens et al., 2016; Voets et al., 2025). It is, however, evident that there are variations in how activation maps are statistically thresholded in clinics and research. Even if dynamic thresholding is advised, precise consensus is difficult to achieve. Deciding on the threshold will depend on the expertise of the involved professionals. Moreover, the surgical question and the proximity of pathology to active brain areas might drive adjustments of thresholds. McKinsey et al. (2010) might provide some comfort to the problem with thresholding: an experienced clinician set t-thresholds to individual patients and individual tasks and tested the reproducibility over four tasks (reversed visual checkerboard, text listening, antonym word generation, and finger tapping) by adjusting the individualized thresholds by ±20%. They did not find that the wider thresholding affected the reproducibility significantly, suggesting that confidence in thresholded activation maps can be found within a range of values. Ni et al. (2019) was the only study to adapt paradigms for patients with variations in cognitive abilities, which is reported to be performed in a minority of the epilepsy centers in Europe as well (Bargalló et al., 2020). Adapting language fMRI paradigms for patients’ cognitive abilities, for specific deficits, as well as patient preference, has been proposed for maximizing successful performance and ensuring robust language maps (Voets et al., 2025). Thus, mapping of patients can be optimized by adapting tasks and quantifications to individuals, at a cost of increased reliance and resource demands on each professional involved in running fMRI sessions and analyzing the data from the sessions.

LIs were used for determining laterality by 35% of the responding European epilepsy centers and centers across the world (Bargalló et al., 2020; Benjamin et al., 2018), but assessing language laterality both with and without LIs is requested frequently (e.g., by 100% of institutions in Benjamin et al. (2018)). The reason there is not a greater percentage of centers using LI is possibly related to the lack of consensus on the calculation of LI (Vingerhoets et al., 2023). In this review, 50% of the studies used LIs for their investigations. Bargalló et al. (2020) also found that 24% of the centers use a ROI analysis to determine activation in clinical practice, which corresponds relatively well with the 33% of the included studies that applied ROIs to determine language activations. Thus, even if the reviewed studies were heterogeneous and few, the general outtakes from them survive in light of reports from clinics, which is likely related to the heterogeneity in clinical applications.

### 4.4 Limitations

Systematic reviews are inherently limited by the databases searched and the search criteria used. This can result in relevant studies being excluded. For example, a paper by Gonzálvez et al. (2016) met the inclusion criteria but was identified through a manual review of a citation in another paper (Voets et al., 2025) after the systematic search was defined and finalized. Thus, it was not introduced into the results in order to preserve the methodological rigor of the approach and avoid undermining the reproducibility of the findings. The omission highlights a potential limitation to the search, indicating that there may be other studies that could have provided additional evidence to the review. Moreover, the small selection of studies and the heterogeneity of the populations and study designs in the studies makes it difficult to directly compare results and therefore limits the strength of the conclusions.

#### 4.4.1 Limitations to tb-fMRI

Accurate delineation of brain regions subserving functions of interest using tb-fMRI typically relies on contrasts between activation and baseline conditions within a given paradigm. The specific configuration of these contrasts may vary depending on factors such as study objectives, feasibility constraints, and the availability of validated paradigms. This variability is reflected in the studies included in the present review. Nevertheless, fMRI enables whole-brain functional mapping, is widely available across clinics, and can be adapted for addressing several sensory-perceptual, motor, and cognitive functions that could be relevant to the patient's symptoms and the surgical question (e.g., Benjamin et al., 2018; Lakhani et al., 2023; Pasquini et al., 2023; Voets et al., 2025). FMRI cannot, however, be used to directly determine which areas are critical to a function instead of merely supporting that function, and methodological variations between studies and clinics may reduce interpretability of the involved processes further (unless variations are grounded in sufficient rationales to promote progression in the field). Moreover, fMRI is moderately susceptible to false positives (FP) and false negatives (FN) and interpretation of fMRI activation maps may be further complicated because of anatomic distortion, tumor infiltration, edema, neurovascular uncoupling, and marked neuroplasticity (Bookheimer, 2007; Leone et al., 2025; Pasquini et al., 2023; Silva et al., 2018). Nevertheless, compared to other non-invasive techniques, such as MEG and nTMS, fMRI provides a trade-off between FPs and FNs, whereas nTMS has a lower risk of FNs but higher risk for FPs (particularly for language), and MEG introduces higher risks of FNs and lower risks of FPs, compared to fMRI (with ESM as gold standard reference) (Ellis et al., 2020; Leone et al., 2025). These aspects become relevant as surgeons can have differing needs in terms of the specific areas they would like to isolate and the pathology in question, which places great demands on the methodological precision needed to tease apart brain regions responsible for the functions of interest (i.e., mapping eloquent cortex) from regions involved in processes of less interest.

## 5 Conclusions

Motor, language, and memory are distinct functional domains which intersect, and one system may rely on the integrity of another system to function in full. Furthermore, even if motor, language, and memory functions can primarily be seen as modality-independent, brain operations supporting these functions differ depending on the sensory modality in play. This systematic review aimed to synthesize evidence from studies applying comparable visual and auditory paradigms for presurgical planning, which would provide indications to the suitability of presenting presurgical fMRI paradigms to different sensory modalities.

Few studies met the inclusion criteria (i.e., six). The studies were highly heterogeneous in their aims, samples, and approaches. Thus, even if the findings from this review aligns with tendencies in the presurgical fMRI field, the results should in no instance be taken as definite indications of how clinical fMRI is being used across clinical sites, and the included studies do not provide a complete picture of the comparability and utility of auditory and visual paradigms for preoperative brain mapping using fMRI. The scarce number of studies reporting on the comparability of audible and visual fMRI task versions that are applied pre-surgery, and their respective utilities in a presurgical planning process, indicates that a contemporary knowledge gap exists. This is likely related to the fact that sensory modalities are rarely of primary interest in clinical fMRI reports on complex cognitive functions (i.e., supramodal processes) unless pathologies overlap with the location of the sensory systems. Nevertheless, contemporary presurgical language-fMRI approaches increasingly support individualized paradigm selection based on the surgical objective as well as the patient's sensory, attentional, and cognitive capacities. In this context, auditory and visual paradigms may engage overlapping higher-order, modality-independent language networks while also recruiting modality-dependent processes (e.g., differences in frontal and temporal engagement). Accordingly, they should perhaps not be regarded as entirely interchangeable, but rather as complementary probes of language functioning that may reveal distinct aspects of preserved and impaired processing.

Overall, uncovering both the shared and modality-dependent mechanisms underlying auditory and visual language paradigms may contribute to improving the validity, adaptability, and clinical sensitivity of presurgical language mapping with fMRI. Such considerations may be particularly important in patients with modality-specific sensory, linguistic, and/or other cognitive deficits. The available evidence remains, however, insufficient to draw firm conclusions regarding auditory and visual language paradigms in presurgical fMRI. Thus, even if the direction of the contemporary literature and praxis suggests that the modality choice should be adapted to the patient, the evidence base for doing so is limited.

## CRediT authorship contribution statement

**Fredrik Brekke:** Writing – review & editing, Writing – original draft, Visualization, Validation, Methodology, Investigation, Formal analysis, Conceptualization. **Karsten Specht:** Writing – review & editing, Supervision, Methodology, Funding acquisition, Conceptualization. **Victoria Sherwood:** Writing – review & editing, Supervision, Project administration, Funding acquisition, Conceptualization. **Jakub Wojciechowski:** Writing – review & editing, Formal analysis, Conceptualization.

## Declaration of competing interest

The authors declare the following financial interests/personal relationships which may be considered as potential competing interests:Fredrik Brekke reports financial support was provided by The Research Council of Norway [Grant number **354139**]. Fredrik Brekke reports that financial support was provided by NordicNeuroLab AS [Grant number]. If there are other authors, they declare that they have no known competing financial interests or personal relationships that could have appeared to influence the work reported in this paper.

## Data Availability

Data will be made available on request.
