# Supplementary material for: A systematic review of the impact of stimulus presentation modality on fMRI studies for presurgical mapping
Source: Neuroimage Rep. 2026 Sep 29;6(4):100412. doi: 10.1016/j.ynirp.2026.100412 (PMC13651669; doi:10.1016/j.ynirp.2026.100412)
Supplement: Supplementary file 1 [file mmc1.docx]

**Systematic search strategy: Database search**

Embase <1974 to 2025 April 18>

Ovid MEDLINE(R) ALL <1946 to April 18, 2025>

APA PsycInfo <1806 to April 2025 Week 1>

1	Functional magnetic resonance imaging/	145464

2	limit 1 to english language	143245

3	limit 2 to humans	133322

4	limit 3 to peer reviewed journal	131367

5	limit 4 to "remove preprint records"	128606

6	(fMRI or "functional magnetic resonance imaging" or "functional MRI" or BOLD).ti,ab,kw.	264363

7	limit 6 to english language	259547

8	limit 7 to humans	226875

9	limit 8 to peer reviewed journal	222054

10	limit 9 to "remove preprint records"	219188

11	(audio or auditive or auditory or audible or acoustic).ti,ab,kw,tw.	612323

12	limit 11 to english language	573113

13	limit 12 to humans	404612

14	limit 13 to peer reviewed journal	384246

15	limit 14 to "remove preprint records"	382424

16	(visual or vision or visuospatial or "visuo-spatial").ti,ab,kw,tw. 1841158

17	limit 16 to english language	1716250

18	limit 17 to humans	1358660

19	limit 18 to peer reviewed journal	1304906

20	limit 19 to "remove preprint records"	1300197

21	memory/	323817

22	limit 21 to english language	307780

23	limit 22 to humans	230460

24	limit 23 to peer reviewed journal	214711

25	limit 24 to "remove preprint records"	212755

26	language/	219468

27	limit 26 to english language	205627

28	limit 27 to humans	190752

29	limit 28 to peer reviewed journal	177336

30	limit 29 to "remove preprint records"	176604

31	(motor or motoric or movement or language or memory).ti,ab,kw.	3420421

32	limit 31 to english language	3231391

33	limit 32 to humans	2369876

34	limit 33 to peer reviewed journal	2232207

35	limit 34 to "remove preprint records"	2220903

36	("Pre-surgical" or presurgical or "pre-operative" or Preoperative or Pretreatment or "Pre-treatment" or surg* or guide* or clinical or Rehabilitation or neurosurg*).ti,ab,kw.	19643964

37	limit 36 to english language	17654219

38	limit 37 to humans	14369140

39	limit 38 to peer reviewed journal	14184309

40	limit 39 to "remove preprint records"	14155976

41	25 or 30 or 35	2303324

42	5 or 10	244245

43	15 and 20 and 40 and 41 and 42	509

44	remove duplicates from 43	300
