# Supplementary material for: A systematic review of the impact of stimulus presentation modality on fMRI studies for presurgical mapping
Source: Neuroimage Rep. 2026 Sep 29;6(4):100412. doi: 10.1016/j.ynirp.2026.100412 (PMC13651669; doi:10.1016/j.ynirp.2026.100412)
Supplement: Supplementary file 2 [file mmc2.docx]

**Systematic Review Protocol Overview**

**Problem**: What are the implications of conveying paradigm instructions and task stimuli auditorily and visually for mapping of sensory and functional (motor, language, and memory) processing and production systems in the brain ahead of surgical interventions?

**Focus on presurgical mapping:** The only routine application of fMRI clinically is for presurgical mapping of individual patients, mostly for medically intractable epilepsy and brain tumors (Specht, 2020). Thus, the lens of this review is influenced by the current applications and standards.

**Focus on use of audible and visual tasks:** Differing study features (e.g., stimuli, task, population, imaging hardware) make it difficult to directly compare studies. Instructions and stimuli in fMRI studies are typically conveyed visually. However, information outside of the MR environment rarely involves only a single sensory modality (e.g., vision). During wakeful hours, the brain is constantly exposed to stimuli, which are detected, separated, and, possibly, integrated by our sensory processing systems. Also, the trajectory from sensory perception towards cognitive and behavioural operations might be significantly altered in different patient populations (e.g., due to lesions or prior surgery). Due to these factors, studies with similar parameters that accounts for possible differences and/or interplay between multiple sensory modalities might be of value for instructing praxis withing the presurgical fMRI domain.

**Rationale for conducting the systematic review:** The aim is to investigate the scientific evidence that is available for making decisions about visual and auditory paradigms for presurgical fMRI of motor, language, and memory functions. The end goal is to contribute to improved guidance for clinical brain mapping with auditory and visual task-based fMRI, which might have implications for patients with/without visual impairments.

**Inclusion and Exclusion**

Articles must meet the following demands to be **included** in the final sample after screening:

- Equivalent auditory and visual fMRI tasks must be applied.
- The fMRI tasks must be used for presurgical brain mapping.
- One (or more) of the following functions must be addressed with the tasks: Motor, memory, or language.
- Population: Adolescents and adults.

Articles are to be **excluded** from the final sample after screening if:

- They are basic research articles that are *not* used for presurgical mapping, despite using comparable audible and visual paradigms on clinical groups.
- This means that other types of interventions are to be excluded from the sample.

- They are basic research articles applying auditory and visual paradigms instructing future use for neurosurgical brain mapping.
- These might be saved for theoretical purposes (i.e., introduction and discussion) but do not meet the exact criteria.

- They are used for diagnostic purposes that are not presurgical (e.g., diagnosing disorders of consciousness (DoC)).
- Resting-state fMRI

Paper types to be excluded: Not peer reviewed, thesis defence papers, conference abstracts (but if relevant, then they can be checked for full text paper that later has been published), pre-prints, non-English language, full text not available, duplicate, theoretically irrelevant (see above for problem and definition of inclusion/exclusion criteria).

**Review protocol:** PRISMA.

**Search strategy:**

**Medical search-engine databases:**

- EMBASE (Emtree / MeSH)
- MEDLINE/PubMed (MeSH)
- APA PsycInfo

Search strings:

(fMRI or “functional MRI” or “functional magnetic resonance imaging” or BOLD).ti,ab,kw.

(motor or motoric or movement or language or memory).ti,ab,kw.

("Pre-surgical" or presurgical or "pre-operative" or Preoperative or Pretreatment or "Pre-treatment" or surg* or guide* or clinical or Rehabilitation or neurosurg*).ti,ab,kw.

(audio or auditive or auditory or audible or acoustic).ti,ab,kw,tw.

(visual or vision or visuospatial or "visuo-spatial").ti,ab,kw,tw.

Ti. = Title

Ab. = Abstract

Kw. = Keyword

Tw. = Text word

**Thesauri (MeSH, Emtree):**

Medical Subject Headings (MeSH) terms are specifically designed for MEDLINE. APA PsycInfo adds MeSH to those articles that are indexed in PubMed (MEDLINE) as well. EMBASE also includes MeSH words but is based on another controlled vocabulary structure called “*Emtree*”, which will be used for functional magnetic resonance imaging in Ovid, since it is indexed there but not in MeSH. It is not an issue that EMBASE and APA PsycInfo are not entirely dedicated to MeSH. Word search is used for finding specific terms that occur in literature but are not indexed in either MeSH or Emtree.

- Controlled vocabulary usage: Standardization of terms. Connects terms to various wordings associated with a concept (e.g., “Functional magnetic resonance imaging” to “fmri” and “blood oxygenation level dependent signal”).
- Exploding terms (Term hierarchies): Selected terms + all narrower terms connected to them are detected and integrated to the search.

Example:

- Symbol in search string with explode function = memory/
- Abbreviation/code for MeSH term only = .mh.
- Explode + weighted search on the specific word = *memory/

**PICO framework:**

- **Population:** Patient candidates for surgery
- **Intervention:** Task-based (preoperative) fMRI
- **Comparison:** Auditory and visual paradigms used to equivalently measure functions related to motor, language, and memory.
- **Outcome:** Brain activity, which may be reported in terms of localization (including sensitivity and specificity), laterality, surgical outcomes, and concordance with other methods (e.g., Electrical stimulation mapping (ESM)).

**Quality Assessment of Methodology:**

Two independent reviewers will rate the quality of each article. Disagreements will be resolved through discussion or consultation with a 3rd reviewer.

**Reporting results**

Key messages should be communicated in a manner that is accessible to experts and non-experts. The report of results should be described simply to increase readability independently of the reader’s background. Findings need to be reported transparently and should be connected to the defined problem and topic.

**Categories:**

- Study: Authors, Year
- Participants: Sample size (sex), Patient pathology (e.g., Epilepsy), Age, Handedness/Hemispheric lateralization, lesion site.
- Aims: For example, assessing whether an auditory task version adds to a standard clinical fMRI protocol with vision-based paradigms.
- Task characteristics: Active/control conditions, cognitive domain and specific functions, modalities, preparation (training, instructions) and pre-fMRI assessment.
- Measurement/analysis characteristics: MRI acquisition (e.g., field strength), threshold (e.g., p-value corr/uncorr, t-threshold, fixed/adaptive), data preprocessing, analyses.
- Main findings: Laterality (index), brain regional activations, sensitivity and specificity, success/outcome.

**Systematic review management platform:** Rayyan.AI
